# Organic Room-Temperature Polariton Condensate in a Higher-Order Topological Lattice

**DOI:** 10.1021/acsphotonics.4c00268

**Published:** 2024-08-08

**Authors:** Christoph Bennenhei, Hangyong Shan, Marti Struve, Nils Kunte, Falk Eilenberger, Jürgen Ohmer, Utz Fischer, Stefan Schumacher, Xuekai Ma, Christian Schneider, Martin Esmann

**Affiliations:** † Institute of Physics, School of Mathematics and Science, Carl von Ossietzky Universität Oldenburg, 26129 Oldenburg, Germany; ‡ Institute of Applied Physics, Abbe Center of Photonics, Friedrich Schiller University Jena, 07743 Jena, Germany; § Fraunhofer-Institute for Applied Optics and Precision Engineering IOF, 07743 Jena, Germany; ∥ Max-Planck-School of Photonics, 07743 Jena, Germany; ⊥ Department of Biochemistry, University of Würzburg, 97074 Würzburg, Germany; # Department of Physics, Center for Optoelectronics and Photonics Paderborn (CeOPP), and Institute for Photonic Quantum Systems (PhoQS), Paderborn University, 33098 Paderborn, Germany

**Keywords:** fluorescent protein, Su–Schrieffer–Heeger model, microcavity exciton polariton, bosonic condensation, higher order topological insulator

## Abstract

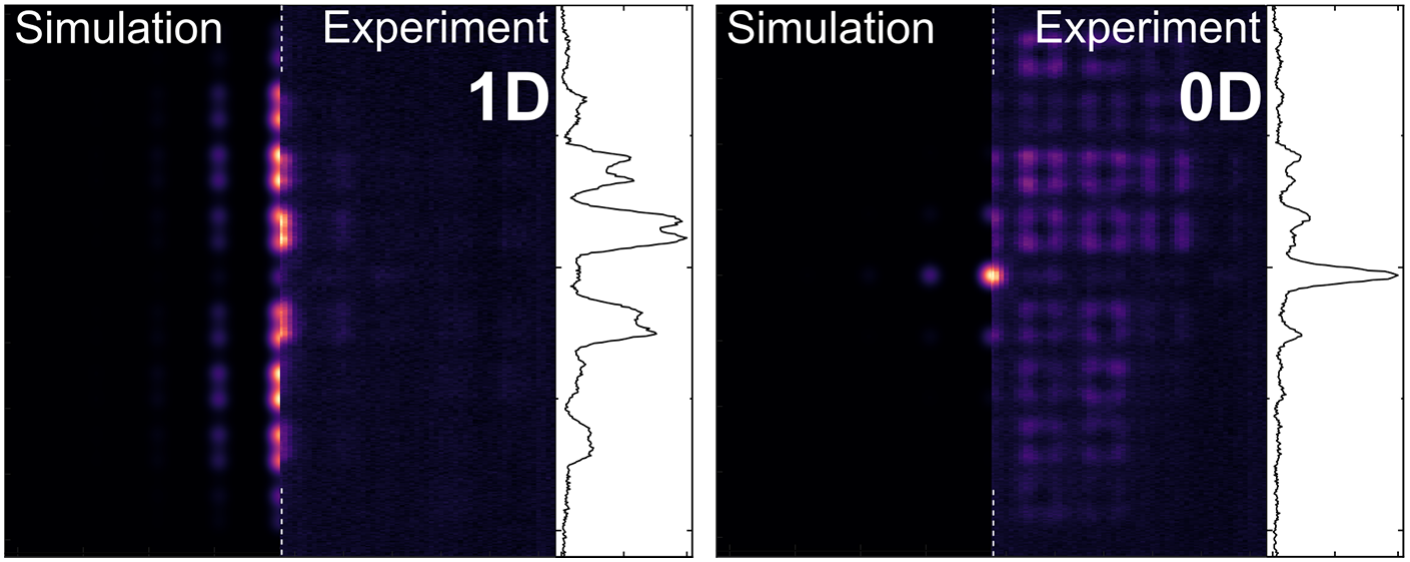

Organic molecule exciton-polaritons in photonic lattices are a versatile platform to emulate unconventional phases of matter at ambient temperatures, including protected interface modes in topological insulators. Here, we investigate bosonic condensation in the most prototypical higher-order topological lattice: a 2D-version of the Su–Schrieffer–Heeger model. Under strong optical pumping, we observe bosonic condensation into both 0D and 1D topologically protected modes. The resulting 1D macroscopic quantum state reaches a coherent spatial extent of 10 μm, as evidenced by interferometric measurements of first order coherence. We account for the spatial mode patterns resulting from fluorescent protein-filled, structured microcavities by tight-binding calculations and theoretically characterize the topological invariants of the lattice. Our findings pave the way toward organic on-chip polaritonics using higher-order topology as a tool for the generation of robustly confined polaritonic lasing states.

## Introduction

Organic molecules hosting tightly bound Frenkel excitons are a particularly promising and easily accessible platform for the realization of cavity exciton-polariton devices.^1−4^ The exciton Bohr radius similar to the size of a single molecule yields binding energies on the order of 1 eV, making these excitons stable at ambient conditions. Polariton lasing^5,6^ in organic matter was first demonstrated in polymers^3^ followed by other molecules.^2^ Fluorescent proteins are a more recent addition to this group of low-cost, flexible, active materials for room-temperature polariton lasing and Bose–Einstein condensation.^7−11^ They feature excellent quantum efficiency and, due to their structure consisting of an exterior β-barrel around an interior chromophore, their emission wavelength is widely tunable at virtually identical chemical properties.

Engineering of the spatial profile of polariton condensates is most commonly realized by structuring the photonic cavity, e.g. by plano-concave photonic potentials that confine the optical field in all three dimensions of space.^8^ Based on these advances, the formation of room-temperature polariton condensates in coupled 1D arrays of spherical cap-shaped, plano-concave optical cavities in topologically trivial and nontrivial arrangements has been demonstrated.^10^

Employing nontrivial topology of the photonic band structure to control the confinement of polaritonic modes at the interface between topologically different domains is particularly appealing for the generation of robust polariton lasers at room-temperature on chip. While lasing from 0D topological defects in a 1D bulk (i.e., a first order topological insulator) has been observed in various organic and inorganic polariton systems,^10,12,13^ only very recently a first experimental demonstration of second-order topological polariton lasing (i.e., lasing from a 0D corner mode embedded in a 2D bulk) was discussed.^14^ However, the preservation of long-range coherence of exciton-polaritons in the topologically protected one-dimensional channels which emerge in a second-order topological insulator at well-designed interfaces has not been observed thus far.

Here, we employ organic polaritons with excitons in the fluorescent protein mCherry^15^ coupled to a 2D lattice of plano-concave microcavities hosting topological 1D and 0D defects. Under pulsed optical pumping, we observe polariton lasing from both types of protected states. We locally pump a macroscopic 1D polariton condensate into the 1D protected state and observe a resulting spatial extent of the condensate of 10 μm along the 1D channel which we prove by spatially mapping the *g*^(1)^ coherence in an interferometric measurement. These measurements are accompanied by a detailed theoretical characterization of the nontrivial bulk topology of our lattice geometry. The results further advance the field of on-chip polaritonics toward topologically robust control over polariton lasers with multiple dimensionalities.

## Results

**Figure 1:**
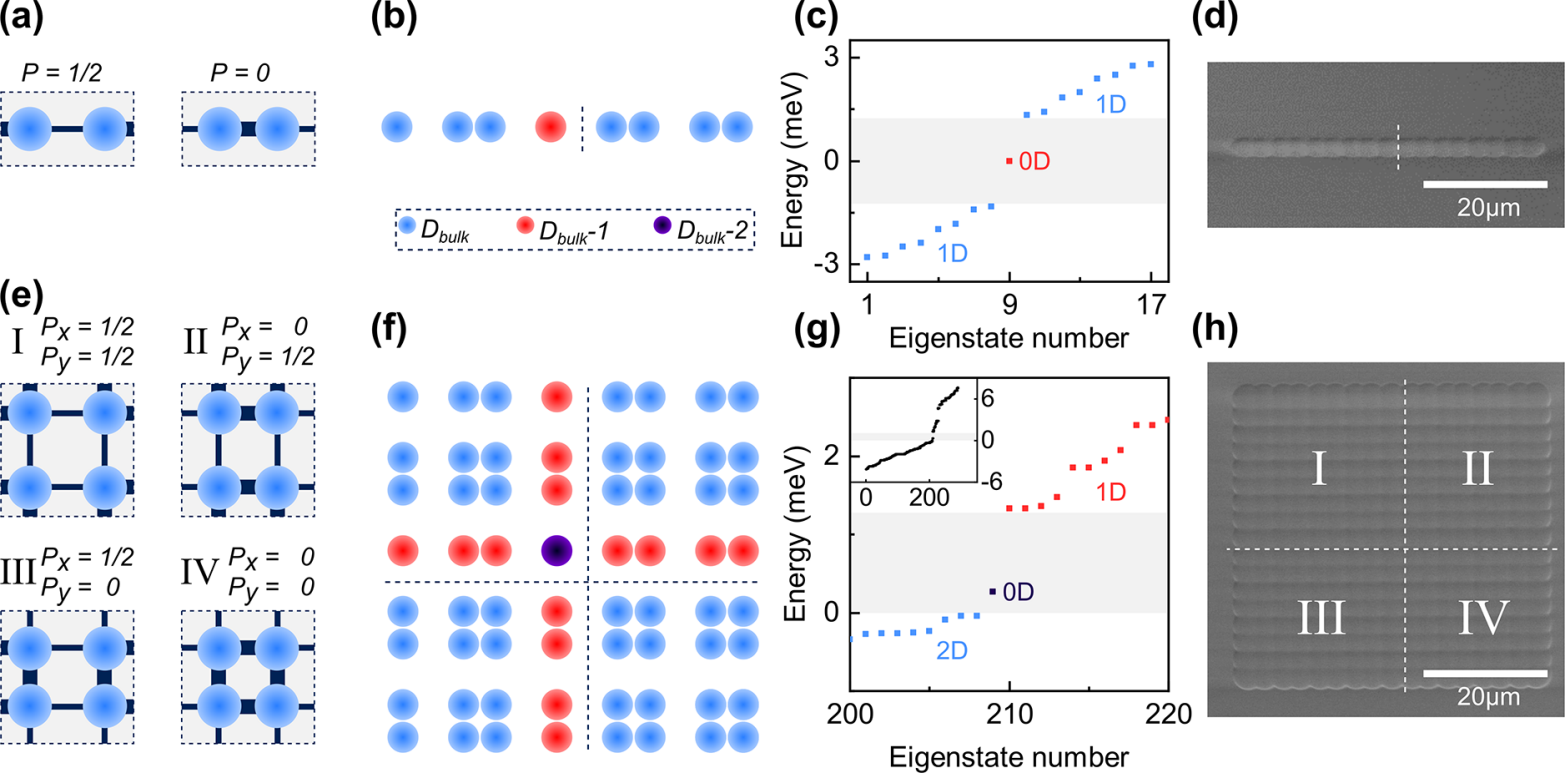
1D and 2D implementations of the SSH model. (a,e) Unit cells in 1D (a) and 2D (e). A topologically nontrivial phase supporting protected edge modes arises if the intercell hopping is larger than the intracell hopping. Thick (thin) lines indicate strong (weak) hopping. (b,f) Sketch of the implementations as used in the experiments for a 1D SSH chain (b) with a 0D defect (red) and of a 2D SSH lattice (f) with line defects (1D, red) and a monomeric defect (0D, violet). Dashed lines mark the interfaces between domains of different bulk polarization *P*_*x*/*y*_ calculated via the Wilson-loop approach. (c,g) Calculated tight-binding eigenenergies of the 1D (c) and 2D lattice (g) for 2 meV strong and 0.9 meV weak hopping. The 1D case shows a 0D gapped defect state. The 2D case (bottom) shows dispersive gapped 1D states and a gapped 0D state since an additional next-nearest neighbor (NNN) hopping of *a* = 2 meV is included. The color of the plotted eigenstates represents the dimensionality with respect to the bulk. In (c) red corresponds to the monomeric defect, while in (g) red represents the 1D states and violet the 0D state. (d,h) Scanning electron microscope (SEM) images of the 1D and 2D photonic lattice implemented by producing indentations in the shape of spherical caps with *d* = 4 μm in one distributed Bragg reflector (DBR) of the organic microcavity via ion beam lithography.

Figure 1 illustrates the topologically different phases of the Su–Schrieffer–Heeger (SSH) model in one (panel (a)) and two dimensions (panel (e)). For a dimerized lattice composed of centro-symmetric unit cells, a topologically nontrivial phase arises in a single band, one particle, tight-binding approximation if the intracell nearest-neighbor hopping integral *w* is smaller than the intercell hopping *v*. In the 1D case, this nontrivial topology can for example be expressed via the winding number of the chiral momentum-space Hamiltonian in the basis of the spin-1/2 Pauli matrices.^16,17^ By virtue of the bulk-boundary correspondence, a finite 1D chain in the nontrivial phase exhibits localized states at the monomeric termination points interfacing the topologically trivial vacuum. These states decay exponentially into the bulk and exhibit complete sublattice polarization, i.e. the wave function of the topological interface mode has nonzero components on only one sublattice of the SSH chain. Due to the chirality of the Hamiltonian, the eigenenergy spectrum is symmetric and thus the defect state remains pinned at the bandgap center, protected from any fluctuations that do not break chirality.

The (nontrivial) topology of a 2D lattice can be characterized by its bulk polarization. We calculate this quantity via the well-established Wilson-loop approach^14^ (Supporting Information Section S1). In the present case, the polarization acquires *P* = 1/2 (*P* can only be 1/2 or 0) for a dimension along which the lattice exhibits nontrivial topology while it is *P* = 0 otherwise. This results in four possible unit-cells in the 2D case as shown in Figure 1e, with nontrivial topology along none, one, or both directions of space.

Figure 1b,f illustrates our specific implementations of the lattice geometries in one and two dimensions. In the 1D chain, we interface a topologically nontrivial phase on the left with a topologically trivial phase on the right. The dashed line marks the domain boundary while the red site labels the resulting topological monomeric defect, which is one dimension lower that the embedding bulk. In 2D, we interface the four possible topologically different unit cells as sketched, resulting in two 1D line-defects (red, one dimension lower than the bulk) forming a 0D monomeric defect at their intersection (violet, two dimensions lower than the bulk). Note that in ref (14) the lattice is composed only of unit cells of type (I), while our implementation constitutes a distinctly different 2D extension of the original 1D SSH model with distinctly different values of the topological invariant associated with it.

We perform effective tight-binding simulations of the two structures resulting in the eigenenergy spectra plotted in Figure 1c,g. Parameters were 2 meV for the strong and 0.9 meV for the weak hopping links. For the 1D case, we retrieve the well-established cosine-shaped band with a topological band gap around zero energy due to the staggered hopping and the gapped topological mode at zero-energy. For the 2D case, it has been shown that if only isotropic nearest-neighbor hopping is included^14,18^ the spectrum remains symmetric in energy but no global band gap around zero is present such that any state with a dominant amplitude contribution on the 0D defect can couple to energetically (near-)degenerate bulk modes. This degeneracy may be lifted through at least two effects: (1) The inclusion of a NNN hopping amplitude *a*(14) (cf. Supporting Information Section S1) that represents additional diagonal hopping links between nontrivial unit cells. (2) A small anisotropy in the hopping links, i.e. *v* and *w* being slightly different along *x* and *y*.^18,19^ For the theoretical calculation in Figure 1g we included a NNN hopping of *a* = 2 meV^14^ resulting in a globally gapped 0D state slightly above zero energy (violet symbol).

In Figure 1d,h, we show SEM images of the 1D and 2D photonic lattices implemented following the concept in Figure 1b,f by producing arrays of coupled plano-concave organic microcavities. The image shows overlapping indentations in the shape of spherical caps with diameter *d* = 3–5 μm and 77–155 nm depth, which we fabricate into a glass substrate via ion beam lithography. Hopping links correspond to a center-to-center distance between neighboring indentations of 0.65*d* for the weak and 0.52*d* for strong links. The indentations serve as a template for depositing a SiO_2_/TiO_2_ DBR with 8 pairs and a center of the optical stop band at 610 nm (see Methods section for details). A solution of the fluorescent molecule mCherry is then laminated between this structured DBR and a second, planar DBR to form a lattice of resonant, plano-concave microcavities (see Methods Section and Supporting Information Section S4 for details).

**Figure 2:**
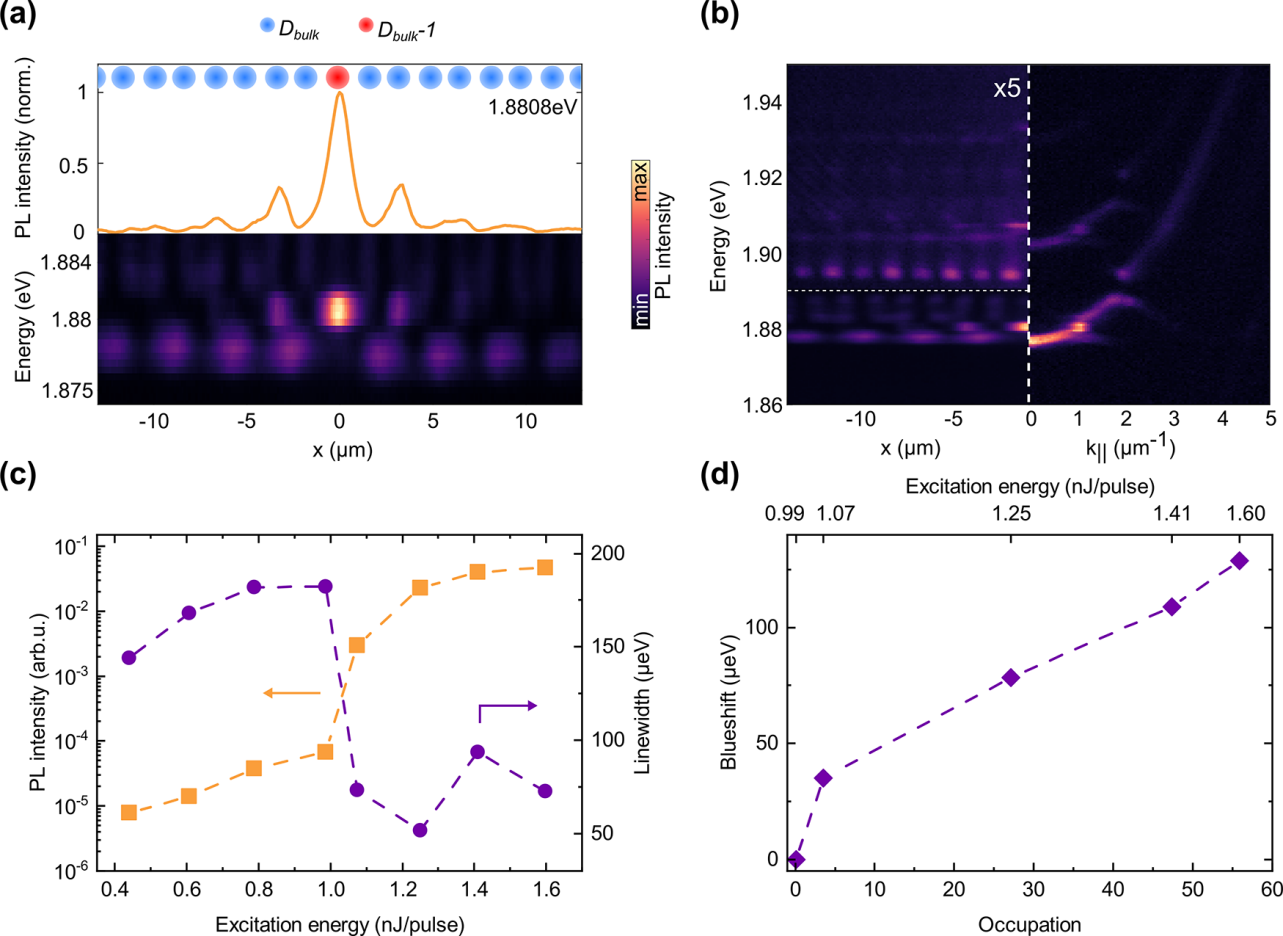
1D SSH chain. (a) Bottom: Energy-resolved real-space photoluminescence (PL) under nonresonant cw excitation at 532 nm. The spectrally gapped, topologically protected 0D mode is exponentially localized on the central topological defect, with a measured sublattice polarization of *S* ≈ 0.72. Top: Spatial cross-section extracted from the bottom panel around 1.88 eV. (b) Real space (left) and momentum- (right) resolved PL spectra of the 1D SSH chain. The dispersive s-band features a topological gap with the defect mode localized in momentum space at the edge of the first Brillouin zone (*k*_∥_ ≈ 1 μm^–1^). Further bands arise from p-orbitals along *x* and *y*. For better visibility, the upper left part above the dashed white line has been multiplied by a factor of 5. (c) Input–output characteristic (orange) of the 0D edge defect under pulsed nonresonant excitation (532 nm, 7 ns pulse duration). A clear nonlinear threshold behavior is observed at pulse energies around 1.04 nJ, accompanied by a step-like collapse of the emission line width (violet) to the instrument resolution limit around 70 μeV. At even higher pulse energies, the emission shows typical saturation behavior. (d) Blueshift of the PL above lasing threshold. Beyond an average polariton occupation of ⟨*n*⟩ = 2, we find a shift of ∼1.7 μeV per polariton.

As a first step, we analyze the optical behavior of a single line segment of the 2D lattice, which is a one-dimensional SSH chain featuring a central domain boundary as shown in Figure 1 (top row). Figure 2 shows experimental PL measurements on a 1D SSH chain of mCherry filled plano-concave microresonators. Under nonresonant cw excitation at 532 nm localized on the central topological defect, we record the energy-resolved real-space PL map in panel (a, bottom). Two Bloch bands separated by a topological band gap around 1.88 eV arise from the (anti)symmetric hybridization of s-type Laguerre-Gaussian orbitals localized in each microresonator. Based on a coupled oscillator model fitted to the measured polariton dispersion of a planar microcavity^8^ (see Supporting Information Figure S1) we deduce a vacuum Rabi splitting of 210 meV and a red-detuning of 220 meV from the exciton at 2.1 eV for the band structure in Figure 2. Within the topological band gap resides the topologically protected interface mode, for which we show a cross-section in the upper part of the panel. We measure an exponential decay length into the bulk of ∼5 μm on the intensity level and a sublattice polarization of  obtained by measuring PL intensities *I*_A_ and *I*_B_ on the A and B sublattices for the two unit cells bounding the topological interface. By changing from real- to momentum-space, we obtain the PL dispersion relation in Figure 2b (right) shown side by side with the real space equivalent (left). The full data set is shown in Supporting Information Figure S4. The topological interface mode is localized at the edges of the first Brillouin zone with a band of (anti)binding s-modes below (above). Bands from higher order p-modes both along and across the SSH chain are also visible.

Under quasi-steady state excitation conditions (532 nm, 7 ns pulse duration, centered on the central defect) we record the input–output characteristic of the topological interface state shown in panel (c). For pulse energies above *P*_th_ ≈ 1.04 nJ, we observe a clear nonlinearity, accompanied by a collapse of the polariton line width down to the spectral resolution limit of the optical detection system. After 1.3*P*_th_ the PL intensity saturates. Due to phase space filling, we also observe a blueshift of the polariton emission with pump power (Figure 2d) that amounts to ∼1.7 μeV per polariton in average occupation. Here, we set the average occupation at the threshold to unity and use that average occupation above threshold is proportional to the detected PL intensity. We interpret the sum of these observations as clear signatures of a transition to polariton lasing in the high polariton density regime. The observable kink in the blueshift is a result of the first data point in Figure 2d being below threshold.

This SSH chain constitutes a 1D section of the 2D lattice, the study of which is detailed in the following.

Our optical study on the 2D SSH lattice is carried out via energy-resolved real-space PL mapping of the lattice depicted in Figure 1h via nonresonant cw excitation on the central defect. This mapping is achieved by a tomographic reconstruction of a 2D map from 1D sections analogous to the mapping in Figure 2.

**Figure 3:**
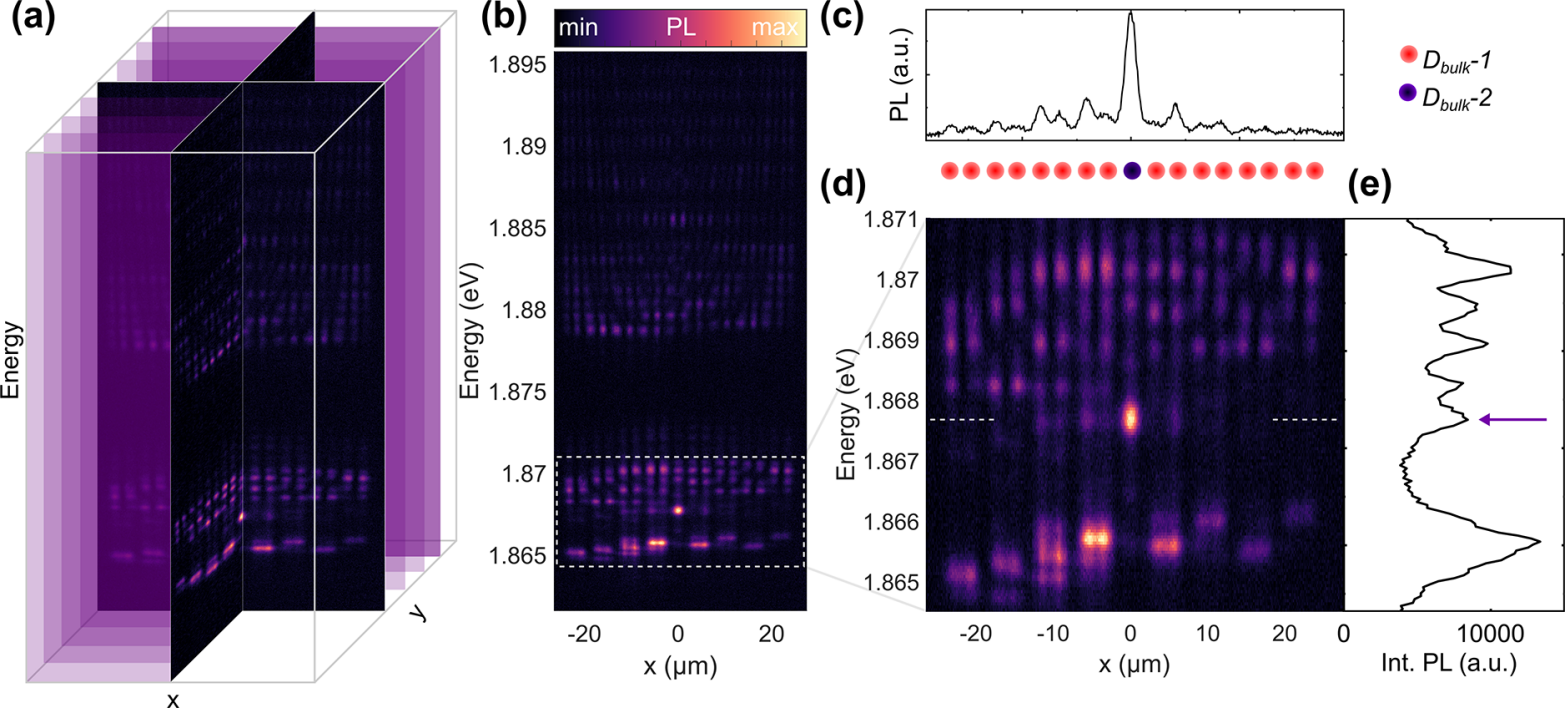
2D SSH lattice: Hyperspectral imaging. (a) Hyperspectral PL spectra as a function of the two spatial coordinates of the lattice. (b) 1D slice through the central 0D defect. The binding and antibinding s-band including the topological 0D state are marked by a dashed white box. (c) Cross-section through the topological state along the white dashed line in (d) showing the sublattice polarization. (d) Zoom-in to the white box in (b). (e) Spatially integrated PL spectrum as an approximation to the density of states corresponding to panel (d). The violet arrow marks the topologically confined 0D state.

In Figure 3a we show spectrally resolved PL of 1D spatial cross sections along one coordinate (*x*) of the lattice. By systematically shifting the detection system along the other spatial coordinate (*y*), we obtain a series of slices that together form a hyperspectral cube containing full spatio-spectral information on the dispersion relation. The spectral data of such a slice containing the topological 0D defect are shown in panel (b). From these data and the zoom-in to the topological gap in panel (d), we assess that the 0D state resides inside a gap close to the lower edge of the band above. In panel (c), we present a spatial cross-section through the 0D state along the dashed white line in panel (d), showing the expected sublattice polarization, further corroborating the expected SSH nature of the state. Figure 3e shows the spatially integrated PL spectrum of the slice in panel (d), i.e. an approximation to the density of states corresponding to this slice, with the spectrally gapped 0D state highlighted by a blue arrow. The state resides close to the upper edge of the topological band gap. Note that such a density of states can either be obtained by momentum-integration over the dispersion relation or—equivalently—by real-space integration over the eigenstates, which we have measured here.

**Figure 4:**
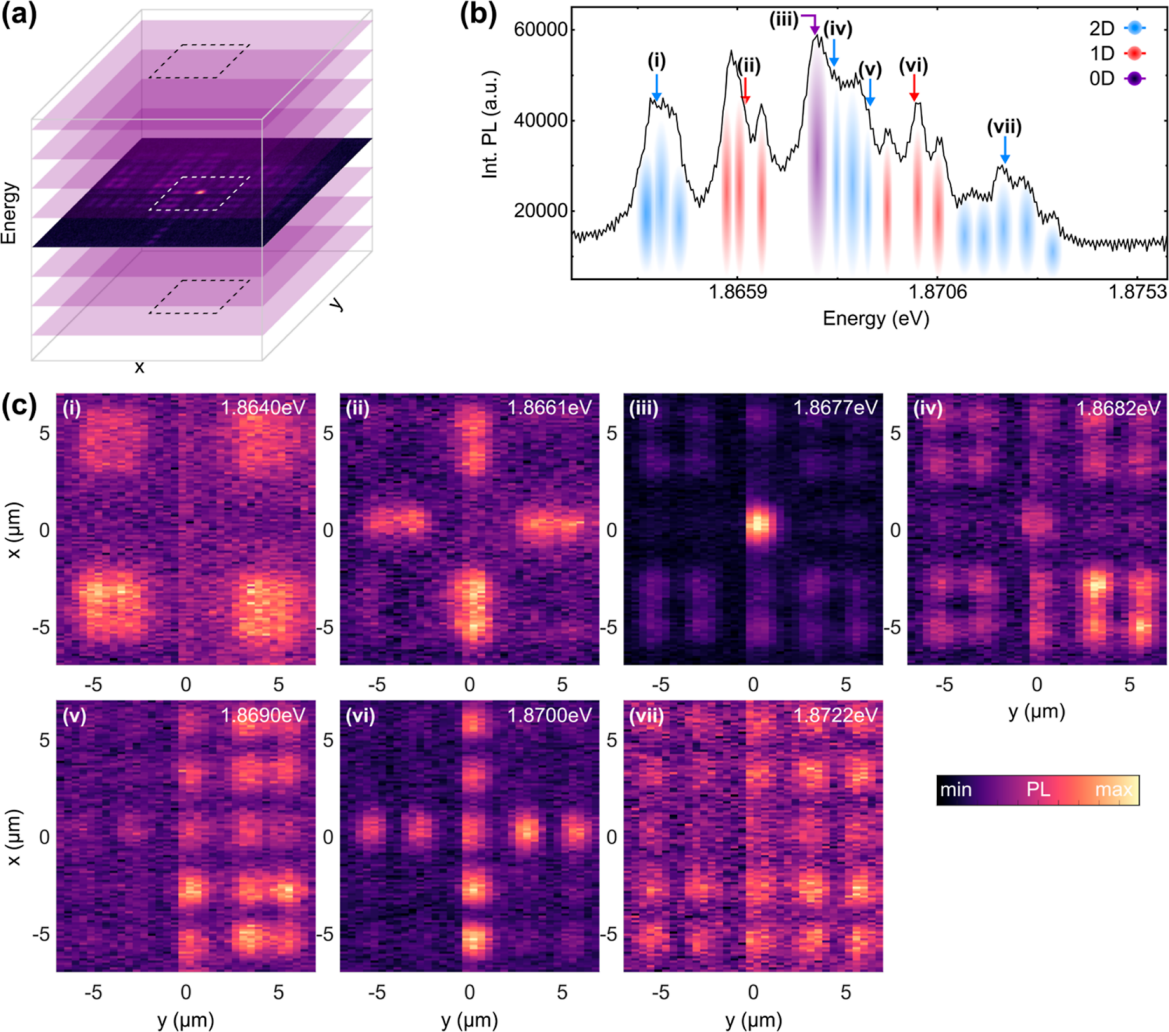
2D SSH lattice: Real space tomography. (a) Extraction of constant-energy spatial maps from the hyperspectral data. The dashed white square marks the area for the zoom-ins in panel (c). (b) Spatially integrated PL spectrum from the central area marked in panel (a). Bands of different dimensionality are color-coded. Arrows mark the spectral positions of the zoom-ins in (c). (c) Constant-energy maps extracted for each of the bands in panel (b). Energies are indicated in the upper right corners. (iii) shows the topologically localized 0D mode, while (i) and (vii) show binding and antibinding bulk states, respectively. (ii) and (vi) show binding and antibinding 1D states.

By taking constant energy slices from the hyperspectral imaging cube as shown in Figure 4a, we obtain real space reconstructions of the PL distribution emerging from individual sub-bands of the 2D SSH lattice. The dashed white in panel (a) marks the area of the lattice for which zoom-ins are shown in panel (c). Panel (b) shows a PL spectrum integrated over the dashed box, as an approximation to the density of states in this area. The most prominent features in this spectrum are color-coded to illustrate the sequence of sub-bands in the s-band of the band structure with their corresponding dimensionality. The energetically lowest sub-band is a binding 2D bulk band (i) as opposed to the antibinding counterpart at the top of the s-band (vii). We furthermore find binding (ii) and antibinding (vi) 1D states extending along the 1D channels intersecting at the center of the lattice, as well as 2D bulk states (iv,v), which are binding in one dimension and antibinding in the other. As before, we find that the 0D state (iii) resides close to the upper edge of the spectral gap.

**Figure 5:**
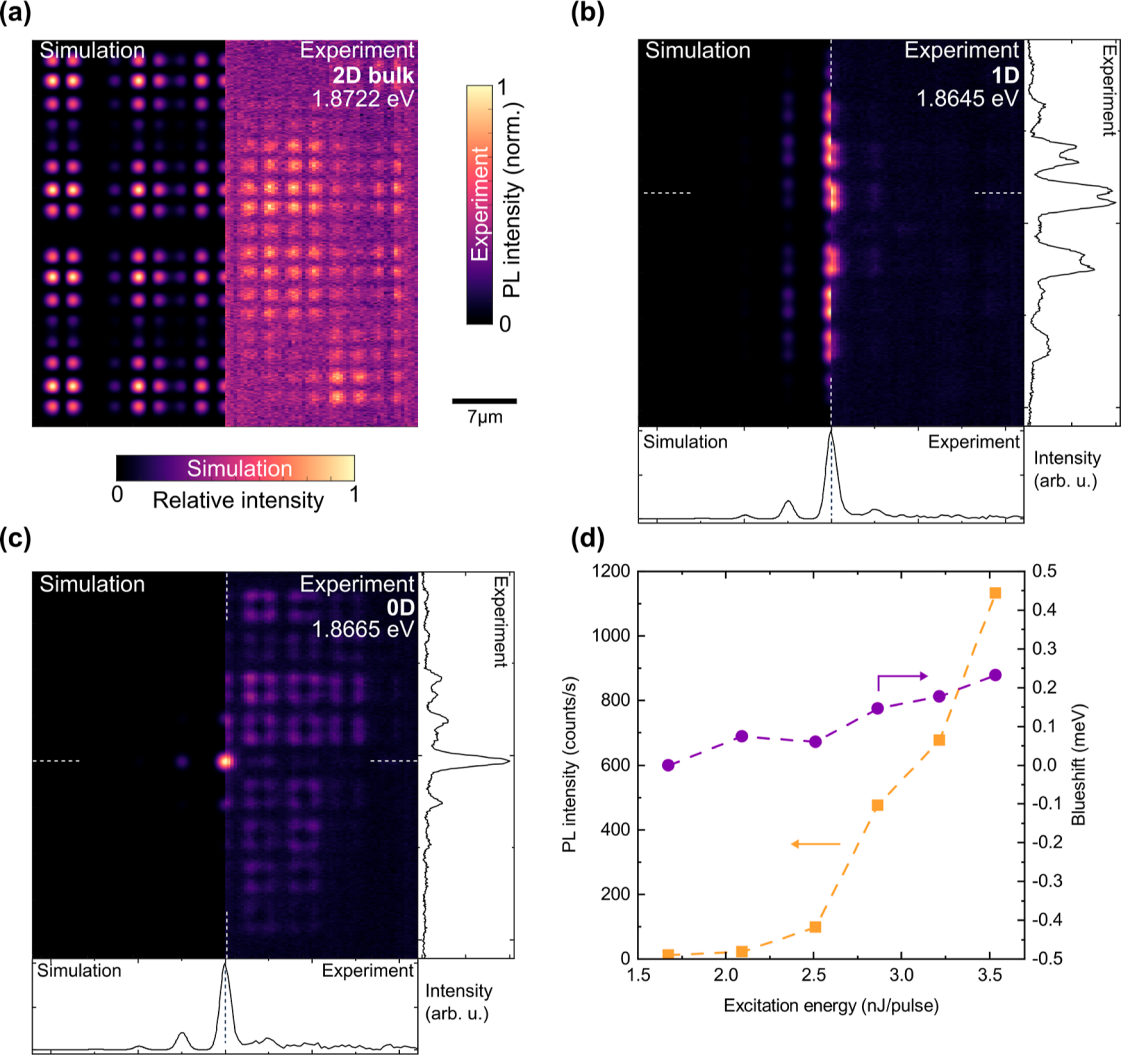
2D SSH lattice: Simulation and lasing. (a–c) Spatial modes of a polaritonic 2D SSH lattice. The left half of each panel shows the spatial intensity distribution of an eigenmode calculated with a tight-binding model including a 3% anisotropy in the hopping links (see Supporting Information Section S3). The right half of each panel shows corresponding experimental PL images of these spatial modes obtained from hyperspectral tomography (see Methods section) taken under local off-resonant cw excitation at 532 nm on the central monomeric defect. Panel (a) shows an exemplary 2D bulk mode (antibinding), (b) shows a topologically protected 1D channel mode and (c) the 0D mode at the central monomeric defect of the lattice. (d) Input–output characteristic (orange) and accompanying blueshift (violet) of the polariton lasing transition of a 2D SSH lattice under off-resonant excitation at 532 nm with 7 ns laser pulse duration localized on the central defect.

**Figure 6:**
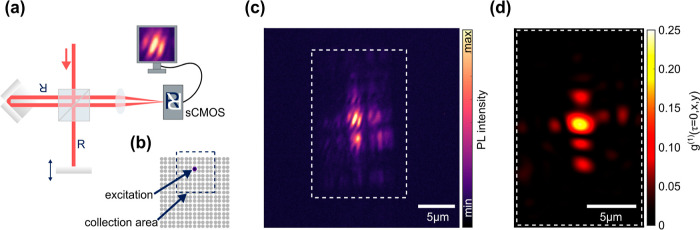
1D polariton condensate in a 2D SSH lattice. (a) Schematic of the Michelson interferometer with a retroreflector in the reference arm, flipping the image along two spatial axes and a flat mirror in the delay arm on a translation stage. (b) Position of the excitation (violet dot) and collection area (dashed square) within the 2D SSH lattice. (c) Corresponding interference image at zero time delay between the interferometer arms. (d) False color representation of the first order correlation function *g*^(1)^(τ = 0, *x*, *y*) extracted from the interference image shown in (c).

In Figure 5a–c we present three constant-energy sections of the hyperspectral data set in the right halves of the panels and compare them to real-space eigenstates from a tight-binding calculation, convoluted with an s-type orbital to achieve the simulated intensity distributions. Zoom-ins to the central regions of these panels are shown as Supporting Information Figure S5. Figure 5a shows a representative bulk state from an antibinding band. Panel (b) shows a topologically protected dispersive, 1D interface state for which we show bosonic condensation extending over multiple lattice sites along the interface (cf. Figure 6). In *x*-direction, this mode exhibits the expected sublattice polarization (see cross-section). Panel (c) shows the higher-order topological 0D monomeric state localized at the central intersection point of the horizontal and vertical line defects. As visible in the cross sections, this state exhibits the expected sublattice polarization both along x and y. To accurately reproduce these experimental findings in the tight-binding simulations, we introduce a 3% anisotropy in the staggered hoppings along x and y,^18^ i.e. *w*_*x*_/*w*_*y*_ = *v*_*x*_/*v*_*y*_ = 1.03 (Supporting Information Section S3). Such an anisotropy most likely arises from the spatial gradient in cavity thickness and leads to a 0D state localized in a global energy gap of 80 μeV width. Conversely, our simulations clearly show that in the absence of both an anisotropy and dominant NNN hopping any monomeric 0D state hybridizes with close-by bulk states and most importantly does not exhibit the expected sublattice polarization of the SSH state.^14^ Our experimental observations of both localization and sublattice polarization thus evidence that the higher-order 0D mode is energetically gapped.

Under nonresonant quasi-steady state excitation (7 ns pulse duration, 532 nm) centered on the central defect, we detect a clear nonlinearity in the input–output characteristic presented in Figure 5d with a threshold of *P*_th_ ≈ 2.5 nJ. The resulting blueshift shown as the inset amounts to 0.2 meV.

The 1D channel modes in the 2D SSH lattice support long-range order of a Bose condensed state in the presence of topological protection. To demonstrate this, we locally pump a polariton condensate into one of the 1D topological channel modes and interferometrically measure its spatial extent by mapping the first order correlation function *g*^(1)^(τ = 0, *x*, *y*) along the channel. This is achieved by the setup sketched in Figure 6a composed of a Michelson interferometer with a retroreflector in one arm tuned close to zero temporal delay. Panel (b) shows the location of the pulsed off-resonant pump (violet circle, 532 nm, 7 ns pulse duration and 3.5 nJ pulse energy) and the area from which we collect the monochromatic emission of the condensate (dashed violet square). Panel (c) shows an image of the polariton condensate emission superimposed with its spatially inverted copy at the interferometer exit. A slight overall offset in wave vector direction between the two copies results in pronounced interference fringes. From this pattern and the emission images of the two arms, we extract^5,20−22^ a spatial coherence map inside the dashed rectangle in (c) and plot it as panel (d). Along the 1D topological edge channel, the coherence extends over a distance of ∼10 μm, i.e. well beyond the size of the localized pump spot of 3 μm diameter. Despite pumping only slightly above threshold, this size is comparable to typical polariton condensates in organic materials under strong pumping.^3,9,23^ This measurement confirms that the polariton emission results from a macroscopically occupied quantum state spontaneously forming coherence extending well beyond the pump volume.

## Conclusion

We have experimentally demonstrated polariton condensation into topologically protected modes of the most prototypical higher-order topological lattice, a 2D-version of the SSH model. This lattice, realized in coupled mCherry-filled DBR microcavities, supports both 0D and 1D topological defect modes, for which we observed pronounced optical nonlinearities, i.e. polariton lasing transitions. In particular, interferometric measurements revealed long-range order in the presence of topological protection via a first-order coherence extending over a length of ∼10 μm in topological 1D channels. A comparison to tight-binding simulations revealed that the observed near-complete sublattice polarization of the 0D state is a clear indication of an energetically gapped higher-order topological interface mode. These findings are an important step toward organic on-chip polaritonics at ambient conditions using higher-order topology as a tool for the generation of robustly confined lasing states and bosonic condensates.

## Methods

The staggered photonic trapping potentials investigated in this work are realized by a structured optical microcavity filled with fluorescent proteins. Figure S3 shows a schematic of the cavity fabrication. First, indentations in the shape of spherical caps, with a depth of 76–155 nm and diameters of *d* = 3–5 μm, are milled into a glass substrate using a focused ion beam (Ga^+^, FEI Helios NanoLab 600i Dual Beam Microscope). The photonic chains and lattices are formed by overlapping the indentations, where the overlap is chosen as a center-to-center distance of 0.52*d* for the strong hopping links and 0.65*d* for the weak hopping links. Next, dielectric mirrors are fabricated by evaporating alternating quarter-wave-layers of SiO_2_ and TiO_2_ on the structured substrate (8 pairs) and a planar substrate (10 pairs), with layer thicknesses chosen such that the resulting DBR stopband is centered around 610 nm. The mirrors are laminated to form an optical microcavity with the cavity volume completely filled by the fluorescent protein mCherry. The protein is prepared in solution of 175 g/L following the recipe described in ref (24) for the protein tdTomato, followed by an additional step of adding 5% vol glycerol at the end of the cleaning process. The cavity is assembled by drop casting 5 μL of the mCherry solution on the patterned DBR and left to dry for 180 s to enhance the lamination properties. The planar mirror is then placed onto the mCherry layer to close the cavity. A weight of 50 g is placed on the cavity and it is left to dry for 48 h at room-temperature in a temperature stabilized environment. This results in cavity lengths ranging from ∼500 to ∼4500 nm.

The experimental setup used for the spectral characterization of the momentum-space of the topological chains is the same Fourier imaging setup as described in Ref.^25^ The structures are excited by an off-resonant continuous wave DPSS laser emitting at 532 nm coupled into the beam path through the reflection pathway of a beam splitter (BS013, Thorlabs) and focused by a microscope objective with an extra-long working distance (Mitutoyo Plan Apo NIR HR 0.65NA) to a spot size of ∼3 μm. The PL emission is collected in reflection geometry by the microscope objective (f1 = 4 mm) through the beam splitter. The back–focal plane of the microscope objective is imaged onto the entrance slit of a spectrometer (Andor SR-500i-A-SIL with an Andor iKon-M 934 CCD camera) using a confocal set of lenses (f2 = 300 mm, f3 = 200 mm, f4 = 300 mm f5 = 400 mm), resulting in emission angle-resolved spectra. The excitation laser is removed in front of the spectrometer by a 550 nm long-pass filter.

For the spectral characterization of the real space emission of the topological chains and lattices, lens f4 is taken out of the beam path, changing the Fourier imaging system into a real space imaging system. The energy resolved spatial emission patterns are recorded by a tomography, for which the last lens in front of the spectrometer (f5) is moved laterally in equidistant steps, thereby imaging different parts of the structure onto the spectrometer slit. We start the scan at the center of the lattice (*y* = 0) progressing to positive values, followed by a scan across the negative halfspace, ending again at the center of the lattice. The images are then stitched from the recorded spectra. For the polariton lasing experiment a Q-switched laser (CNI Laser MPL-III-532–20 nJ) with 7 ns pulse length emitting at 532 nm instead of the continuous wave laser. For the spatial coherence measurement, a Michelson interferometer (as shown in Figure 6a, with retroreflector PS976M-B and beam splitter BSW10R, both Thorlabs) is inserted in the beam path between lenses f3 and f4. After checking for monochromaticity with the spectrometer, the overlapping images from the interferometer are imaged on a sCMOS camera (Zyla 5.5, Andor) to enhance the collection efficiency.

## Supporting Information Available

Supporting Information Document 1 (file type PDF) contains Sections S1–S5. Section S1 describes the topological phases of the 2D SSH mode. Section S2 contains Figure S1 showing strong light-matter coupling in mCherry-filled cavities. Section S3 contains Figure S2 showing tight-binding simulations including hopping anisotropy. Section S4 contains Figure S3 showing a schematic of the cavity assembly. Section S5 contains Figure S4 showing extended real space and momentum resolved photoluminescence spectra and Figure S5 showing Zoom-ins to the central parts of Figure 5. (PDF)
