# Supplementary material for: Organic Room-Temperature Polariton Condensate in a Higher-Order Topological Lattice
Source: ACS Photonics. 2024 Aug 8;11(8):3046–54. doi: 10.1021/acsphotonics.4c00268 (PMC11342920; doi:10.1021/acsphotonics.4c00268)
Supplement: Supplementary file 1 — ph4c00268_si_001.pdf [file ph4c00268_si_001.pdf]

Supporting Information:

Organic Room-Temperature Polariton Condensate in a Higher-Order Topological Lattice

Christoph Bennenhei[1], Hangyong Shan[1], Marti Struve[1], Nils Kunte[1], Falk Eilenberger[2], Jürgen Ohmer[3], Utz Fischer[3], Stefan Schumacher[4], Xuekai Ma[4], Christian Schneider[1], and Martin Esmann[1,]*

[1]Institute of Physics, School of Mathematics and Science, Carl von Ossietzky Universität Oldenburg, 26129 Oldenburg, Germany

[2]Institute of Applied Physics, Abbe Center of Photonics, Friedrich Schiller University Jena, 07743 Jena, Germany; Fraunhofer-Institute for Applied Optics and Precision Engineering IOF, 07743 Jena, Germany; Max-Planck-School of Photonics, 07743 Jena, Germany

[3]Department of Biochemistry, University of Würzburg, 97074 Würzburg, Germany

[4]Department of Physics, Center for Optoelectronics and Photonics Paderborn (CeOPP), and Institute for Photonic Quantum Systems (PhoQS), Paderborn University, 33098 Paderborn, Germany

1. Supplementary section S1: Topological phases of the 2D SSH model

In this section, we theoretically characterize the topologically non-trivial properties of our 2D lattice shown in Fig. 1 (bottom) of the main text. To this end, we decompose the lattice into four domains as shown in Fig. 1 of the main text, and calculate the bulk polarization of the four different unit-cells in these areas. Besides the nearest-neighbor (NN) coupling, the next-nearest-neighbor (NNN) coupling is also included in our work, since the NNN coupling results in a topologically gapped 0D state as previously reported in [1]. The unit-cells can thus be described by the following tight-binding model in k-space

$$\begin{aligned} H &= H_{NN} + H_{NNN} \\ &= \begin{pmatrix} 0 & H_{12} & H_{13} & H_{14} \\ H_{12}^* & 0 & H_{23} & H_{24} \\ H_{13}^* & H_{23}^* & 0 & H_{34} \\ H_{14}^* & H_{24}^* & H_{34}^* & 0 \end{pmatrix} \end{aligned} \tag{1}$$

with NN coupling elements

$$\begin{aligned} H_{12} = H_{34} = J_x + J_x' e^{ik_x a} \\ H_{13} = H_{24} = J_y + J_y' e^{-ik_y a} \end{aligned} \tag{2}$$

Here, $J_{x,y}$ $(J_{x,y}')$ represents the intra-cell (inter-cell) coupling strength along x, y directions, respectively. a is the lattice constant of the unit-cells. Note that in the main text, these notations correspond to $J_{x,y} = w_{x,y}$ and $J_{x,y}' = v_{x,y}$. The elements that denote the NNN coupling, H_{14} and H_{23}, are different for the different unit-cells as listed in Table S1:

Table S1. Next-nearest-neighbor coupling for different unit cells I-IV

	unit-cell I	unit-cell II	unit-cell III	unit-cell IV
$H_{14} =$<br>$H_{23} =$	$J_n e^{i(k_x - k_y)a}$<br>$J_n e^{-i(k_x - k_y)a}$	$J_n e^{-ik_y a}$<br>$J_n e^{-ik_y a}$	$J_n e^{ik_x a}$<br>$J_n e^{-ik_x a}$	J_n<br>J_n

Here, J_n is the NNN coupling strength. The bulk polarization can be calculate by using the Wilson-loop approach [2–5], thus it satisfies the relation

$$P_{x,y} = \frac{1}{2\pi} \sum_j^{N_{bg}} v_{x,y}^j \left(k_{y,x}\right) \tag{3}$$

Here, $v_x^j(k_y)$ is the j-th Wannier center along the x direction in reciprocal space, and $v_y^j(k_x)$ along the y direction. N_{bg} is the total number of the bands below the gap of interest, in our case, there are $N_{bg} = 3$ bands [1] below the first band gap. The Wannier centers can be calculated by solving the eigenvalue problem of the Wannier Hamiltonian

$$H_{W_x}(k_y) = -\ln W_x(k_y), \quad H_{W_y}(k_x) = -\ln W_y(k_x)$$

$W_{x,y}$ are the Wilson-loop operators along the x, y direction in the Brillouin zone, which are defined as

$$W_x(k_y) = F_x(k_x, k_y)F_x(k_x + \Delta k_x, k_y) \cdots F_x(k_x + 2\pi/a - \Delta k_x, k_y),$$
$$W_y(k_x) = F_y(k_x, k_y)F_y(k_x, k_y + \Delta k_y) \cdots F_y(k_x, k_y + 2\pi/a - \Delta k_y),$$

with $[F_x(k_x, k_y)]_{m,n} = \langle u_m(k_x, k_y)|u_n(k_x + \Delta k_x, k_y)\rangle$, $[F_y(k_x, k_y)]_{m,n} = \langle u_m(k_x, k_y)|u_n(k_x, k_y + \Delta k_y)\rangle$ and $\Delta k_x = \Delta k_y = 2\pi/aN$ (here, we choose $N = 500$, i.e. 501×501 points used for describing the first Brillouin zone). The Bloch functions $u_m(k_x, k_y)$ are calculated by using the tight-binding model Eq. (1) and satisfy $\langle u_m(k_x, k_y)|u_n(k_x, k_y)\rangle = \delta_{m,n}$. Therefore, the polarizations of different unit-cells can be numerically obtained and are summarized in Table S2:

Table S2. Polarizations of different unit cells

	v_x^1	v_x^2	v_x^3	P_x	v_y^1	v_y^2	v_y^3	P_y
unit-cell I	0	π	0	$1/2$	0	π	0	$1/2$
unit-cell II	π	0	π	0	0	π	0	$1/2$
unit-cell III	0	π	0	$1/2$	π	0	π	0
unit-cell IV	π	0	π	0	π	0	π	0

The quantized 2D Zak phase is related to the bulk polarization via the relation $\theta = 2\pi P$. The corresponding band gap is trivial when $\theta = 0$, while it is non-trivial when $\theta = \pi$. From the table above, one can see that unit-cell IV is trivial in both directions with $\theta = (0,0)$, while unit-cell I is non-trivial with $\theta = (\pi, \pi)$. Unit-cell III is non-trivial in the x direction and trivial in the y direction with $\theta = (\pi, 0)$, whereas unit-cell II is trivial in x and non-trivial in y with $\theta = (0, \pi)$.

In our experimental configuration as sketched in Fig. 1(b,f) of the main text, we thus describe our lattice as composed of four domains with unit-cells of types I-IV arranged as marked in panels (e,h).

2. Supplementary section S2: Strong light-matter coupling in mCherry-filled cavities

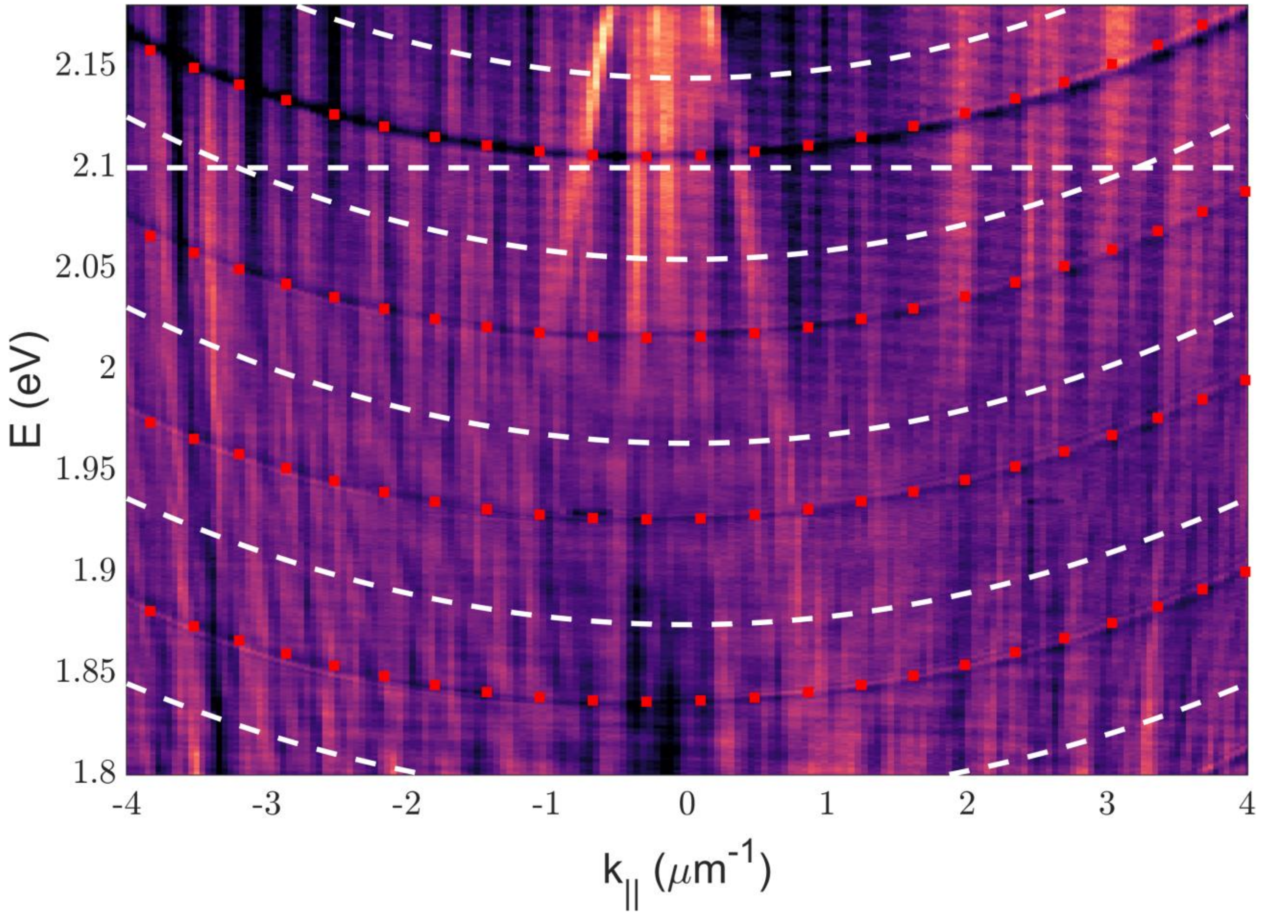

Figure S1. Strong light-matter coupling in mCherry-filled planar microcavity. Polariton dispersion relation measured in white light reflection on a planar microcavity filled with mCherry. A coupled oscillator model (red squares) well accounts for the observed polariton modes with the uncoupled exciton at 2.1 eV in a cavity with $L_{cav} = 4.1$ μm. The model yields a coupling strength of g = 105 meV, i.e. a vacuum Rabi splitting of $\hbar\omega_R = 210$ meV. The dashed white lines mark the uncoupled photonic modes and the uncoupled exciton, respectively.

3. Supplementary section S3: Tight-binding simulations including hopping anisotropy

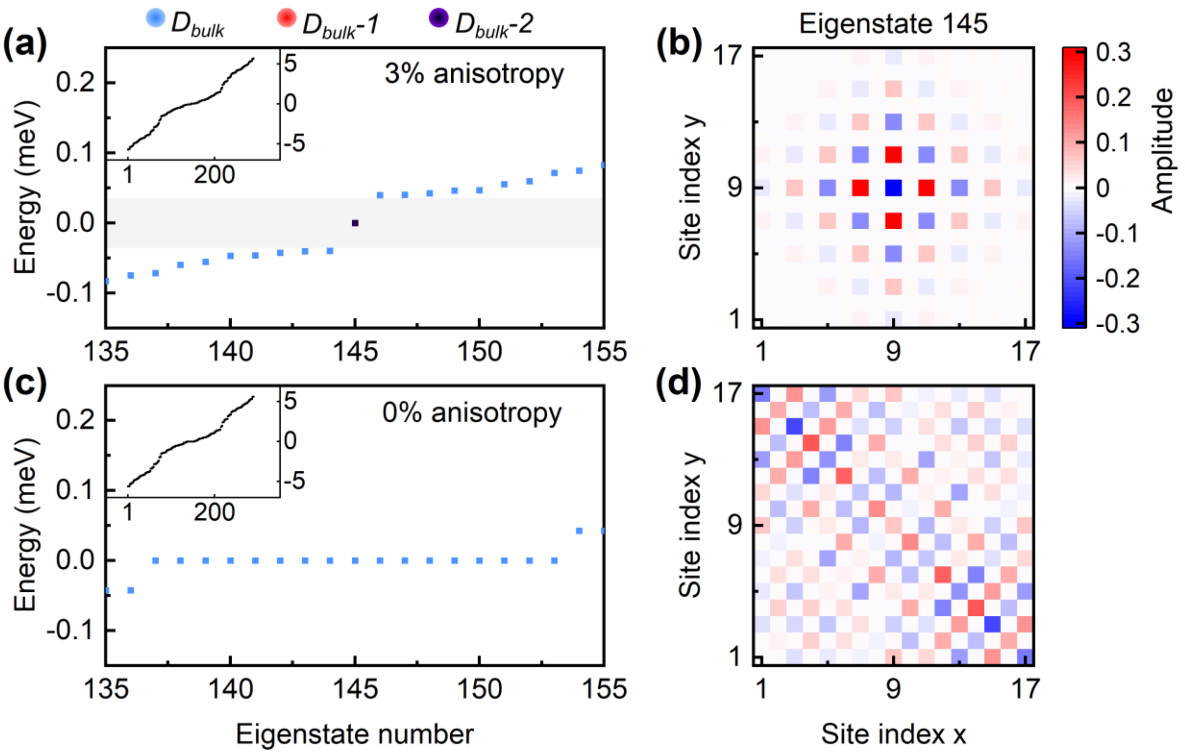

Figure S2. Eigenenergy spectrum of the 2D SSH lattice including hopping anisotropy but no next-nearest neighbor hopping. (a) Eigenenergies of the 2D SSH lattice as shown in Fig. 1 (main text) with $\frac{v_x}{v_y} = \frac{w_x}{w_y} = 1.03$ and 2 meV for the strong and 0.9 meV for the weak hopping elements along the y-direction, i.e. a 3% anisotropy in the hopping terms that is most likely caused by fabrication imperfections. An 80 μeV wide gap opens around zero with one state pinned at its center. While this gap width is below our spectral imaging resolution in the experiment, we may still expect to be able to identify this state albeit with some admixture from close-by bulk states in the signal. Inset: In the absence of next-nearest neighbor hopping the full eigenspectrum is symmetric with respect to zero energy. The simulated states plotted in Fig. 5 of the main text correspond to eigenstates with numbers 145 (0D), 226 (1D) and 28 (2D bulk) in the spectrum shown in Fig. S2(a). (b) The state in the gap shows the expected sub-lattice polarization of the SSH state, is localized at the monomeric central defect and decays exponentially into the bulk. (c-d) For comparison, when hopping anisotropy is switched off, a large number of bulk states around zero energy become near-degenerate and no gap is present.

4. Supplementary section S4: Cavity assembly

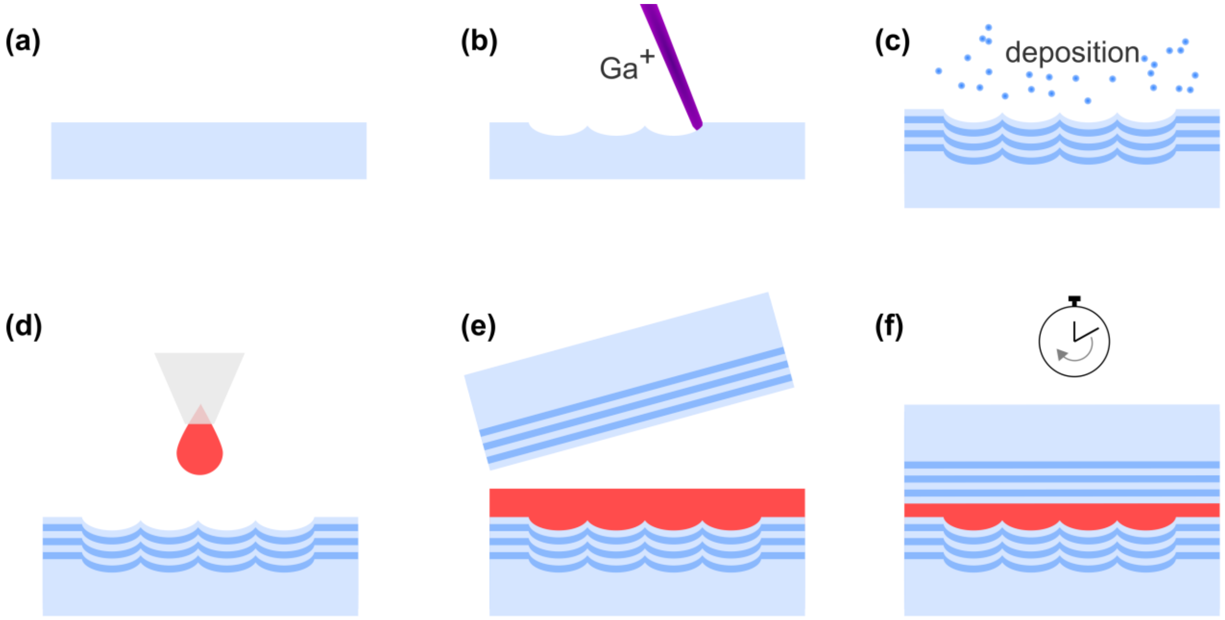

Figure S3. Schematic of cavity assembly. (a) Planar substrate of eco – thin glass (SCHOTT). (b) Indentations in the shape of spherical caps with a depth of d = 155nm are fabricated into the substrate using focused Ga^+ ion beam milling. (c) Alternating layers of SiO_2 and TiO_2 are evaporated onto the patterned (8 pairs) and planar substrate (10 pairs) to create dielectric mirrors. (d) A volume of 5 μL of a 175 g/L fluorescent protein mCherry solution is drop-cast onto the patterned dielectric mirror. (e) A planar mirror is placed onto the mCherry-covered mirror. (f) The laminated cavity is left to dry for 48 h in a temperature-stabilized environment at room-temperature, with a weight of 50 g placed on the top of the mirrors.

5. Supplementary section S5: Additional experimental data

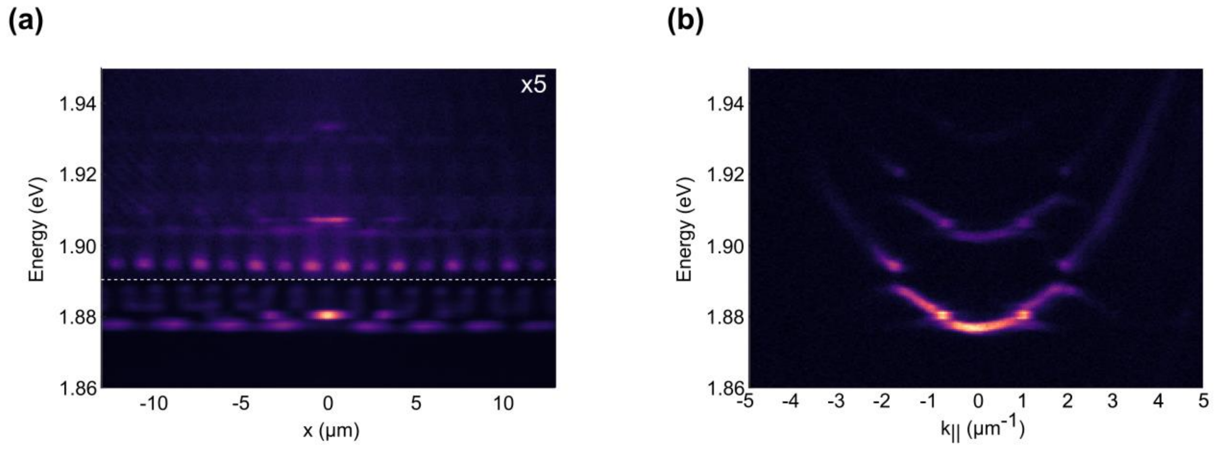

Figure S4. Real space (a) and momentum- (b) resolved photoluminescence spectra of the 1D SSH chain shown in Fig. 2 of the main text. The dispersive s-band features a topological gap with the defect mode localized in momentum space at the edges of the first Brillouin zone ($k_{\|} \approx \pm 1\ \mu m^{-1}$). Further bands arise from p-orbitals along x and y. For better visibility, the upper part of panel (a) above the dashed white line has been multiplied by a factor of five.

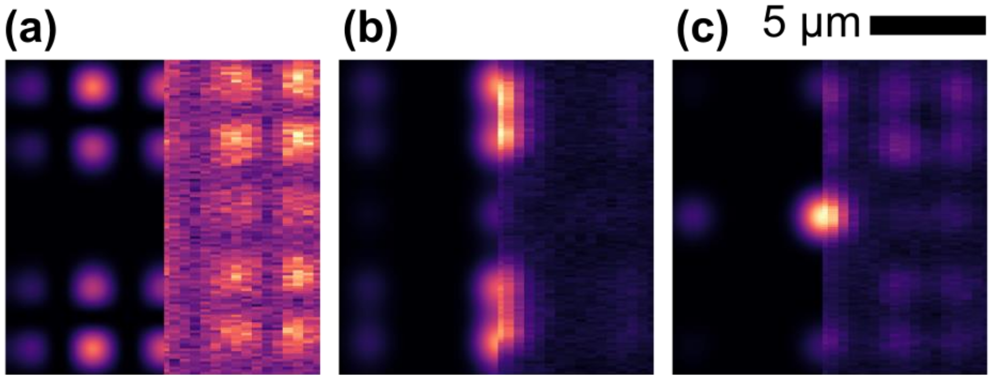

Figure S5. Zoom-ins to the central region of the PL maps (right half of each panel) and their simulated counterparts (left halves) of Fig. 5 (a-c) in the main text.

REFERENCES

1. J. Wu, S. Ghosh, Y. Gan, Y. Shi, S. Mandal, H. Sun, B. Zhang, T. C. H. Liew, R. Su, and Q. Xiong, "Higher-order topological polariton corner state lasing," Sci. Adv. **9**, eadg4322 (2023).
2. W. A. Benalcazar, B. A. Bernevig, and T. L. Hughes, "Quantized electric multipole insulators," Science **357**, 61–66 (2017).
3. W. A. Benalcazar, B. A. Bernevig, and T. L. Hughes, "Electric multipole moments, topological multipole moment pumping, and chiral hinge states in crystalline insulators," Phys. Rev. B **96**, 245115 (2017).
4. B.-Y. Xie, H.-F. Wang, H.-X. Wang, X.-Y. Zhu, J.-H. Jiang, M.-H. Lu, and Y.-F. Chen, "Second-order photonic topological insulator with corner states," Phys. Rev. B **98**, 205147 (2018).
5. M. Kim and J. Rho, "Topological edge and corner states in a two-dimensional photonic Su-Schrieffer-Heeger lattice," Nanophotonics **9**, 3227–3234 (2020).
